# Underexplored and growing economic costs of invasive alien trees

**DOI:** 10.1038/s41598-023-35802-4

**Published:** 2023-06-02

**Authors:** Romina D. Fernandez, Phillip J. Haubrock, Ross N. Cuthbert, Gustavo Heringer, Melina Kourantidou, Emma J. Hudgins, Elena Angulo, Christophe A. Diagne, Franck Courchamp, Martin A. Nuñez

**Affiliations:** 1 Instituto de Ecología Regional, Universidad Nacional de Tucumán-CONICET, CC. 34, 4107 Yerba Buena, Tucumán Argentina; 2 Department of River Ecology and Conservation, Senckenberg Research Institute and Natural History Museum Frankfurt, Clamecystr. 12, 63571 Gelnhausen, Germany; 3 CAMB, Center for Applied Mathematics and Bioinformatics, Gulf University for Science and Technology, Hallawy, Kuwait; 4 Faculty of Fisheries and Protection of Waters, South Bohemian Research Center of Aquaculture and Biodiversity of Hydrocenoses, University of South Bohemia in České Budějovice, Zátiší 728/II, 389 25, Vodňany, Czech Republic; 5 Institute for Global Food Security, School of Biological Sciences, Queen’s University Belfast, Belfast, BT9 5DL UK; 6 Department of Ecology and Conservation, Institute of Natural Sciences, Universidade Federal de Lavras - UFLA, Lavras, Minas Gerais 37200-900 Brazil; 7 Nürtingen-Geislingen University (HfWU), Schelmenwasen 4-8, 72622 Nürtingen, Germany; 8 Department of Sociology, Environmental and Business Economics, University of Southern Denmark, Degnevej 14, 6705 Esbjerg Ø, Denmark; 9 Department of Biology, Carleton University, Ottawa, ON K1S 5B6 Canada; 10 Estación Biológica de Doñana (CSIC), Avda. Americo Vespucio 26, 41092 Sevilla, Spain; 11 Université Paris-Saclay, CNRS, AgroParisTech, Ecologie Systématique Evolution, 91190 Gif sur Yvette, France; 12 Department of Biology and Biochemistry, University of Houston, Houston, TX USA

**Keywords:** Ecology, Environmental sciences

## Abstract

The high ecological impacts of many invasive alien trees have been well documented. However, to date, we lacked synthesis of their economic impacts, hampering management actions. Here, we summarize the cost records of invasive trees to (*I*) identify invasive trees with cost information and their geographic locations, (*II*) investigate the types of costs recorded and sectors impacted by invasive trees and (*III*) analyze the relationships between categories of uses of invasive trees and the invasion costs attributed to these uses. We found reliable cost records only for 72 invasive trees, accumulating a reported total cost of $19.2 billion between 1960 and 2020. Agriculture was the sector with the highest cost records due to invasive trees. Most costs were incurred as resource damages and losses ($3.5 billion). Close attention to the ornamental sector is important for reducing the economic impact of invasive trees, since most invasive trees with cost records were introduced for that use. Despite massive reported costs of invasive trees, there remain large knowledge gaps on most invasive trees, sectors, and geographic scales, indicating that the real cost is severely underestimated. This highlights the need for further concerted and widely-distributed research efforts regarding the economic impact of invasive trees.

## Introduction

Biological invasions are a major component of global environmental change^1^. Ecological, social, and economic impacts of invasive alien species are ever-increasing, and are compounded by other global environmental stressors, such as climate and land use changes^2–4^. Thousands of plant species have been introduced beyond their native ranges and some of them have become invasive in their non-native ranges, with noticeable impacts to the environment and to human well-being^5,6^.

The introduction and movement of alien plant species within and among ecosystems is often intentional, mostly motivated by commercial trade and the ecosystem services they impart^6–8^. Several tree species have been introduced for ornamental purposes, to provide food and construction materials, as well as to mitigate deforestation, desertification, soil erosion, and even climate change^6,7,9,10^. Several of these alien tree species are used for commercial exploitation (e.g., timber and tanning products), therefore contributing to regional, national, and local economies, and are sometimes perceived as beneficial, when they improve social and economic well-being^11,12^. However, past evidence has shown that the benefits associated with the introduction of alien trees were eroded when these species become invasive^13,14^.

The competitive dominance of invasive alien trees often leads to the formation of mono-specific stands in invaded areas, which reduce local biodiversity, change species composition, displace native vegetation and affect native wildlife habitats^15–19^. Many invasive alien trees also alter important ecosystem services such as fire regimes, nutrient, and water cycling^10,18,20,21^, and negatively impact areas of high conservation value^6,21^. In addition, some invasive alien trees are known for driving significant economic losses associated with their excessive water use and significant management expenditure, triggered, for instance, through long-term control programs^14,22–26^.

The fact that some invasive alien trees can simultaneously provide ecological and economic benefits while causing negative impacts generates conflicts between different stakeholder groups, due to their differences in value systems, perceptions, and interests^8,12,27–29^. For this reason, the management and effective control of invasive alien trees remains a major challenge^8,30^. Due to the lack of precautionary risk assessments for many alien trees introduced for different economic uses in some countries^30^ and the steady increase in the number of alien trees listed as invasive globally^7,31^, this pressing problem has a staggering potential to grow. One reason for this ongoing challenge is the limited understanding of the socioeconomic impacts for several invasive trees and specifically the lack of a standardized process for assessing their impacts across different dimensions, which in turn undoubtedly limits efficient control and management actions^32^. Many studies have already assessed the cost records of prominent groups of invasive alien species at different temporal, spatial, taxonomic and other scales^33–35^. Although the impacts of invasive trees are often massive and the economic consequences of their invasion can be high, to date there is no assessment specifically focused on the economic cost of invasive trees.

In order to bridge this knowledge gap, we provide the first synthesis of the economic cost records of invasive alien trees. We aim to (*I*) identify which species in which geographic locations possess cost information, (*II*) investigate which type of cost was recorded and which sectors were impacted, and (*III*) analyze the relationship between categories of uses of invasive trees and the invasion costs attributed to these uses.

## Results

### Cost sources, taxonomic groupings and geographic regions

According to the available data, cost records associated with invasive alien trees between 1960 and 2020 accumulated to a total of $19.2 billion (n = 2886 expanded entries), with an average of $313.89 million per year. About 25% of this total cost was empirically observed ($5 billion, n = 2183), whereas the remainder was associated with potential cost (i.e., not necessarily incurred, but extrapolated or expected). Most of the total cost originated from highly reliable sources ($12 billion, n = 2704). Additionally, the vast majority of the observed costs was deemed to be highly reliable ($4.7 billion, n = 2119). The remainder of the results considers our robust subset that includes only ‘observed’ and ‘highly reliable’ costs.

**Figure 1:**
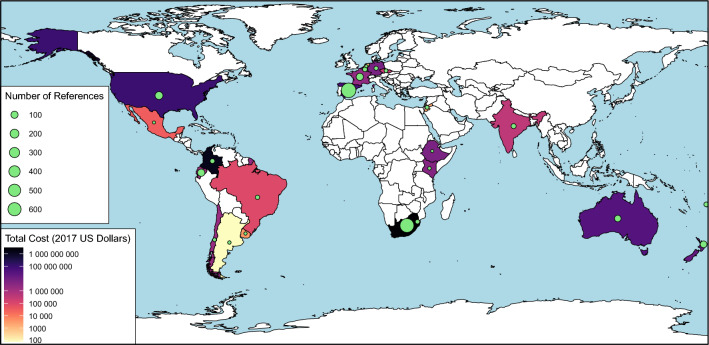
Spatial distribution of recorded costs of invasive trees. The map shows with a color categorical gradient the countries with the highest (black) to lowest (cream) cost recorded (‘observed’ and ‘highly reliable’ costs; 2017 US Dollars) of invasive trees. Countries without observed cost reports are in white. The map also indicates the number of references reporting costs of invasive trees by country with the green node size.

Regarding the spatial distribution of costs of invasive trees, the greatest share of observed costs were reported from South Africa (US$ 3.1 billion, entries n = 697), Colombia (US$1.4 billion, entries n = 18), the United States (US$ 98.0 million, n = 137), Spain (US$ 58.8 million, n = 785), and Australia (US$ 33.5 million, entries n = 74) (Fig. 1). Argentina (US$ 80.5, entries n = 3) had the lowest documented costs. No costs were reported from several countries, mainly in Africa and Asia (Fig. 1). While Spain had by far the most cost records (entries n = 785), it had only moderate total costs reported (US$ 58.8 million). In contrast, Belgium and Ethiopia ranked 8th and 9th in terms of costs, while having very few records each (US$ 6.3 million, entries n = 17, and US$ 6.0 million, entries n = 1, respectively).

**Figure 2:**
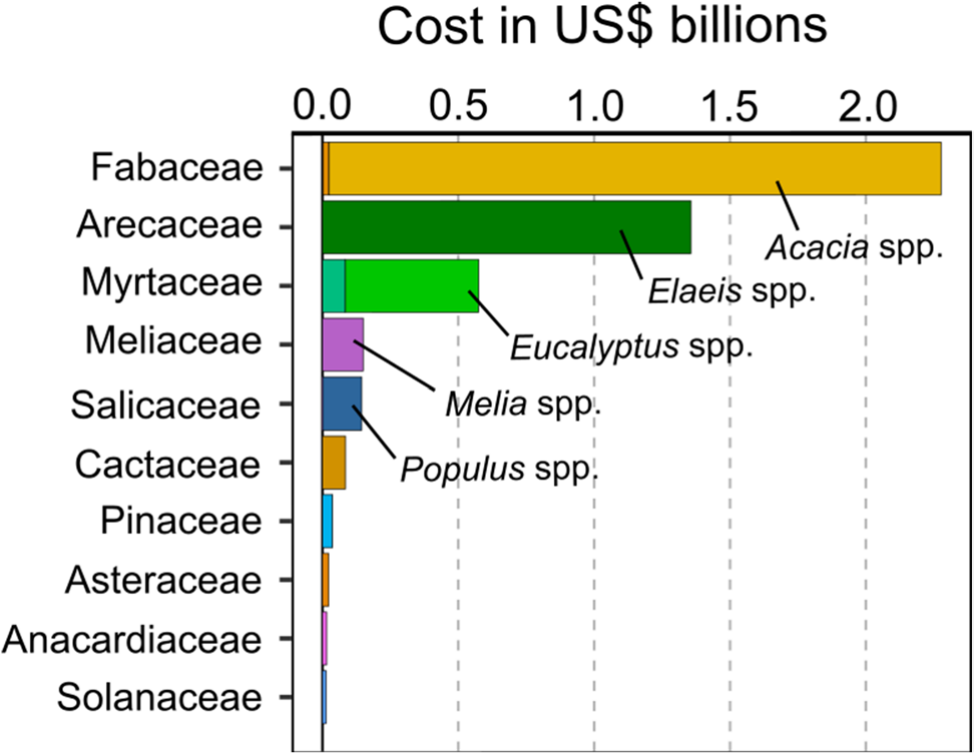
Costliest families and genera. Top ten costliest families of invasive trees and the respective costliest genera of the top five families considering highly reliable, observed costs records (in 2017 US$ billions). Different colors indicate different families and shades distinguish genera.

We found 72 invasive alien tree species (17% of the total number of known invasive trees^31^) including 52 genera and 32 families with costs that were deemed highly reliable and observed. The top ten costliest families of invasive trees in decreasing order were: Fabaceae, Arecaceae, Myrtaceae, Meliaceae, Salicaceae, Cactaceae, Pinaceae, Asteraceae, Anacardiaceae, and Solanaceae (Fig. 2). The cost was very unevenly distributed among the top 10 costliest invasive alien trees, with almost 90% of these costs derived from the invasion of *Acacia mearnsii* (black wattle) and *Elaeis guineensis* (African oil palm) in South Africa and Colombia, respectively (Table 1).

**Table 1:** Top ten costliest invasive alien trees considering highly reliable, observed costs records and their expanded database entry numbers.

Species			Cost ($ millions)	Database entries (n)
Scientific name	Common name	Family		
*Acacia mearnsii*	Black wattle	Fabaceae	2,175	107
*Elaeis guineensis*	African oil palm	Araceae	1,356	18
*Melia azedarach*	Chinaberry	Meliaceae	150	50
*Cereus jamacaru*	Queen of the night	Cactaceae	85	37
*Melaleuca quinquenervia*	Paperbark tree	Myrtaceae	78	23
*Baccharis halimifolia*	Groundseltree	Asteraceae	23	90
*Schinus terebinthifolia*	Brazilian pepper tree	Anacardiaceae	16	9
*Solanum mauritianum*	Tobacco tree	Solanaceae	14	51
*Prunus serotina*	Black cherry	Rosaceae	14	31
*Acacia saligna*	Coojong	Fabaceae	13	21
Total			3924	437

The vast majority of costs of invasive alien trees were caused by terrestrial species ($4.6 billion, n = 1989). However, the semi-aquatic species *Melaleuca quinquenervia* (paperbark tree) in North America and South Africa, and *Baccharis halimifolia* (Groundseltree) in France and Australia also led to substantial costs ($0.1 billion, n = 109).

### Socioeconomic sectors and cost types

**Figure 3:**
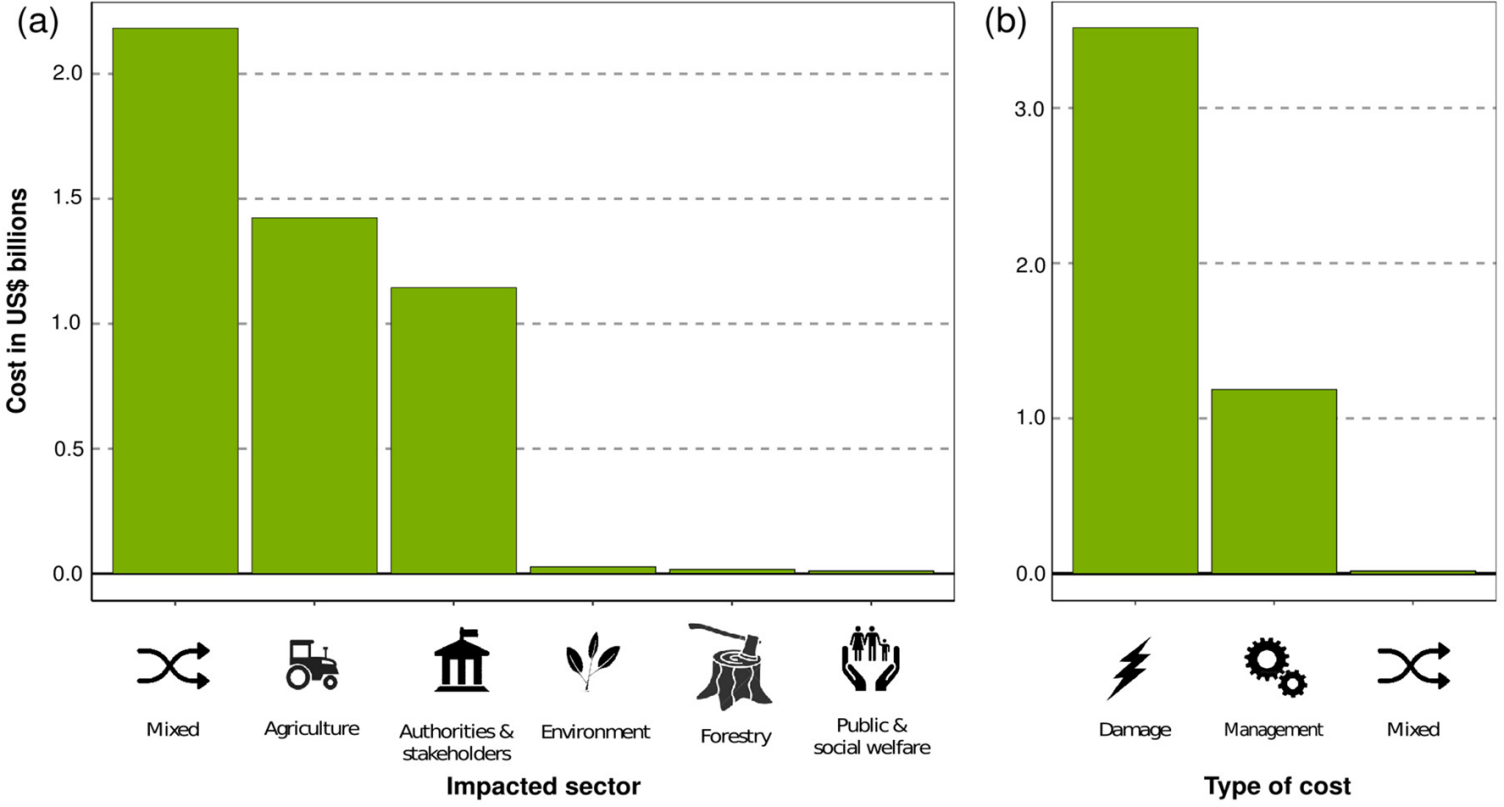
Sectors affected and type of cost. Total costs (highly reliable and observed) of invasive alien trees according to (**a**) impacted sector and (**b**) type of cost.

The sector bearing the greatest cost was agriculture ($1.4 billion, n = 76), followed by authorities-stakeholders (i.e., governmental departments and/or official organizations, $1.1 billion, n = 1952). Other sectors (environment, forestry, and public and social welfare) received ≤ $0.01 billion in costs, while $2.1 billion was incurred by mixed sectors (Fig. 3a). The largest shares of invasive alien tree costs were incurred from resource damages and losses, despite very low numbers of cost entries ($3.5 billion, n = 38). Management spending was less than half of the total damages, but with far more cost entries ($1.2 billion, n = 2070). The remainder of entries consisted of mixed costs for which the relative damage and management cost could not be distinguished ($0.01 billion, n = 11) (Fig. 3b). Most of the management cost was spent reactively on post-invasion actions ($1.1 billion), whereas a much smaller part (< $0.01 billion) was spent proactively, on pre-invasion management or on knowledge funding. At the family level, Fabaceae ($2.2 billion) and Arecaceae ($1.4 billion) caused the largest share of damages, whereas Myrtaceae ($0.6 billion) led to the greatest management expenditure.

### Uses of alien trees

**Figure 4:**
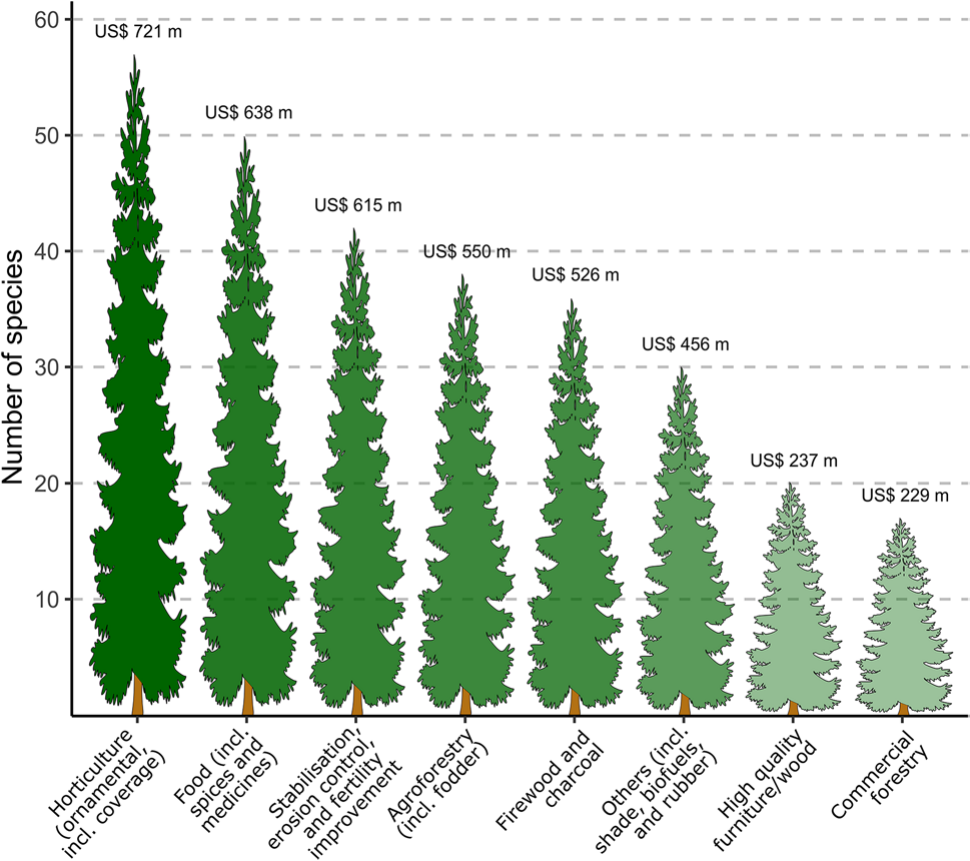
Uses of invasive trees with cost records. Tree height shows the number of invasive tree species for each category of use and the number on top shows the total cost incurred (highly reliable and observed), also indicated by shades of green with darker green representing higher costs (see text for details).

Invasive trees included in InvaCost tended to be those associated with multiple uses, and the numbers of uses ranged from one to eight (Supplementary Table S2 online). The largest share of the $3.97 billion in total species-specific cost entries was attributed to invasive trees that were introduced for multiple uses, with only 14% associated with a single use (Supplementary Table S2 online). Horticultural (ornamental, including coverage) use was the most common among all uses of invasive alien trees (58 species), followed by food (including spice and medicine), stabilisation, erosion control and fertility improvement, agroforestry (including fodder), firewood and charcoal, other (including shade, biofuel and rubber), high-quality timber/furniture and then commercial forestry (Fig. 4). According to this, invasive trees used for horticulture (ornamental, including coverage) and food (including spices and medicines) were found to be the costliest (Fig. 4).

## Discussion

Growing numbers of tree invasions have been reported worldwide, with at least 434 invasive alien tree species linked to ecological and economic impacts^31^. Alarmingly, costs have been reported for less than 20% of these known invasive trees and in limited portions of their invaded ranges, indicating that real costs for most invasive tree species remain unreported. Factors such as uses of different definitions of tree, cost reports without clear identification of the species, reports focusing on invasive tree species which do not necessarily mention the 'invasive' nature of these species (and thus InvaCost search strings not capturing them), lack of cost studies on certain species and regions, and non-monetized costs each contribute to underestimated costs of invasive trees.

Many articles or other sources inferred costs at the genus level, without identifying the species. Indeed, when taking the 191 entries in the InvaCost database for genera that do not identify species, but for which we know to include species listed as "shrub or tree" life form (e.g., *Acacia* spp., *Tamarix* spp., *Ligustrum* spp., and *Prosopis* spp.), the forgone total was between $23.9 billion (using the robust dataset) and $64.7 billion (including low reliability and potential costs). Since our analysis included only entries for genera that include solely tree species (e.g., *Pinus* spp. and *Eucalyptus* spp.), the costs of invasive trees summarized here are again underestimated, and represent a minimum estimate of the total cost.

The Fabaceae (legume) family includes numerous species on the global list of invasive tree species^7^ and bears the highest costs, mainly associated with some *Acacia* species. Most of the *Acacia* species are native to Australia and many of them have been planted around the world for purposes such as forestry, ornamental use, and to provide materials e.g., for furniture^7,14,30^. Although *Acacia* species showed the highest cost records, the number of studies reporting costs for these species was low in comparison with the extensive literature on the ecological impacts and management strategies of *Acacia* species*.*

Like *Acacia* species, several *Pinus* species were introduced internationally and predominantly in the Southern Hemisphere for commercial plantations and agroforestry. Many of these species, such as *P. pinaster*, *P. radiata*, *P. patula*, *P. contorta, P. halepensis* and *P. elliotii*, invade natural ecosystems, displacing native biodiversity, altering fire regimes and nutrient cycles, and decreasing water availability^21,36,37^. Since *Pinus* use for forestry represents the greatest spatial and temporal planting of invasive trees, it was expected that in addition to causing significant ecological damages, these species would be associated with the highest economic costs. Contrary to this expectation, *Pinus* species were not in the list of the top ten costliest invasive tree species in our study. This may be because the perceived benefit of forestry production limits the perceived utility of studying the impacts of *Pinus* plantations. For this or other reasons, such as most of their invaded range being in the Global South, which is generally data-deficient in terms of costs^38–41^, there may be an associated lack of published reports on invasive *Pinus* costs. Additionally, as mentioned above, certain records of pine control costs are indistinguishably mixed with control costs of invasive species that are not trees^26,42^. Other invasive tree species known to possess considerable ecological impacts such as *Prosopis juliflora*, *Tamarix ramosissima, Miconia calvescens,* and *Leucaena leucocephala* also have few recorded economic impacts.

Cost reports are missing from many countries and the countries for which cost records do exist, pertain to only a few invasive tree species. Given the large reporting gaps, it is not possible to make robust inferences on the global representation of the costs related to invasions of tree species. Our results also revealed strong geographic cost unevenness. Specifically, the InvaCost database used in this study contained no invasive tree costs for many countries, particularly in Africa, Asia and to a lesser extent Oceania and Central America. This can likely be partly attributed to the InvaCost database being limited in the number of languages considered for the cost search^39^. Although InvaCost includes cost data searched in over 22 non-English languages^39^, multiple languages still need to be included to improve understanding of costs of invasive trees globally, particularly in Asia. The fact that South Africa is the country with the highest reported cost was expected, given that this country had several invasive tree species reported and importantly, a long history in research and management of invasive species^43,44^, and invasive tree species in particular^45^. On the other hand, the high economic cost reported in Colombia is somewhat surprising, but was mostly related to the invasion by *Elaeis guineensis* (African palm). The African palm was introduced to Colombia in the 1950s, and since then, Colombia has become one of the main producers of palm oil in the world^46^. African palm cultivation contributes significantly to the economy of this country, but at the same time, the control of this species as an invader and, predominantly, the damage in the production of other crops due to its invasion, have resulted in significant economic losses^47^. As is often the case with anthropogenic impacts, those most impacted by invasive palm damage (e.g., smallholder farmers of other crops) are likely not the same individuals who most benefit from the production of palm oil (e.g., large corporations^48^).

It is widely recognized that early detection and management of invasive species has economic benefits-averting damages to various degrees—and therefore often results in lower control costs^49,50^. For invasive trees, we found substantial damage costs, but with reporting delayed compared to management expenditure. This is consistent with a lack of research interest and/or capacity in assessing and/or documenting the costs of these species proactively. In addition, most records of management costs were identified at post-invasion stages; which can indicate the small investments for early detection and management of several invasive tree species, as is the case also for other groups of invasive species^50^, higher costs of reactive responses to invasion^40^ or the difficulty of obtaining this information.

Most alien plant species were intentionally introduced via pathways linked to the plant trade, particularly for ornamental horticulture and forestry, but also for food, agroforestry and medicines^7,51–53^. The plant species with a greater variety of uses are introduced more frequently, which increases their opportunities to invade and cause major problems^7,30,53^. In line with this, we found that most of the invasive trees with cost records were introduced for multiple uses. Particularly, most invasive trees in our dataset were introduced for ornamental purposes as well as for provision of food and medicine. Many of these species are still considered useful in some regions and in some cases, new purposes have since been found, different from those that prompted initial introduction and cultivation, but this may be related to the lack of other plant options available^30^.

In particular, forestry with alien tree species provides a significant economic benefit through the trade of products and employment, but at the same time, these plantations can act as an important bridgehead for future invasions^54,55^. The forestry industry is still planting alien tree species because of the realized economic benefits that come with such investments. However, the ecological, economic, or social costs associated with the abandonment of these plantations and the spread and invasion of these species may counterbalance their benefits^13,27,56^. In addition, they are not borne by the same stakeholders, so the benefits of invasive trees should not be a reason for accepting their costs. Yet, the economic benefits obtained from the use of invasive trees can be important for local economies, and must be taken into account in the formulation of policies^12^ (e.g., subsidies for transitioning to native species). Accordingly, it should be considered that the greatest long-term benefits will be obtained from the species with low risks of causing ecological and economical damage^30^. Similarly, urban forestry is planting large numbers of alien tree species as ornamentals, and these plantations can facilitate invasions for associated species such as invasive insects^57,58^, which can use stressed native and alien urban trees to establish and go on to cause billions of dollars of damage in cities^59^. Beyond recent calls for urban tree planting for climate change adaptation purposes^60^, a healthy urban canopy is necessary for adaptation to future climate change, which will be especially severe in cities that experience the urban heat-island effect^61^. An analogous trade-off must be considered to balance the risk posed by the use of alien trees in cities and the damage they may cause, both to other urban trees, and through escape of alien trees and their pests into surrounding areas.

The control of invasive tree species that have ecological or economic benefits can be a challenge, since for most people ‘green is good’ irrespective of the species, and many people do not easily distinguish invasive trees from native ones^62,63^. There are multiple examples of negative impacts associated with invasive trees that are not perceived as such by citizens due to the prominent benefits associated with these trees and their strong cultural value and charisma^8,64,65^. Thus, preventive actions of these species can be frowned upon by society and even hindered in some cases^64,65^. This highlights the need for stronger and more open communication with stakeholders and communities to understand the benefits they derive from alien tree species, whether other benefits are put at risk by these species, and whether alternative native species are available to fill existing needs^66^. Furthermore, it is necessary to advise stakeholders and communities to seek alternative income sources through native plantations^66^ and/or diversifying local industries, as occurred in South Africa where the outcomes of deliberations with stakeholder are being implemented in policy^41^. These options would help minimize conflicts of interest and reliance on alien trees, which ultimately may help avoid or reduce undesirable invasions.

## Conclusions

Our synthesis shows that, to date, there are few available records of economic costs for invasive trees and only for a small subset of these species (e.g., over 80% of all known invasive trees and large parts of the world have no cost records). Further research is urgently needed to accurately establish the current numbers of invasive alien tree species worldwide, thereby helping to identify knowledge gaps in understanding of their economic impact. Despite limited data being available, our results clearly indicate that invasive trees cause economic losses at the multi-billion-dollar scale. There is a need for more rigorous and consistent reporting of observed/materialized costs for many invasive trees at the species level across their entire invaded ranges. Future studies could consider the abundance of invasive trees and the invaded area in order to obtain better estimates of their economic impacts. We also argue for more comprehensive risk assessments of spread beyond plantation ranges and invasion risk prior to the introduction and spread of any alien tree species introduced for a particular use. Last, it is important to keep in mind that we use costs as a standardized way of studying economic impacts, but invasive trees cause significant problems for biodiversity and ecosystem functioning that are difficult or even impossible to quantify in monetary terms. This should remain a concern and the focus of discussions as to the costs and benefits of these species, and how to navigate associated trade-offs.

## Methods

### Tree definition and data source of their economic costs

There are many definitions of “tree”. In this study, we followed the tree definition agreed on by IUCN’s Global Tree Specialist Group (GTSG): “*a woody plant with usually a single stem growing to a height of at least two metres, or if multi-stemmed, then at least one vertical stem five centimeters in diameter at breast height*”. The GlobalTreeSearch database uses this definition and includes all species that are recorded as naturally growing as a tree somewhere, representing the most comprehensive list of the world’s tree species^67^. Thus, this database includes palms and some cacti. Based on this list, we identify the species with tree growth form in the InvaCost database to obtain the list of invasive trees with records of economic costs.

The InvaCost database compiles reported monetary costs of invasive alien species worldwide in a systematic and standardized manner^34^; version 4.1 available at https://doi.org/10.6084/m9.figshare.12668570, This most up-to-date version-at the time of writing-includes 13,553 cost entries (i.e., rows of unique cost data), which were collected from both English and non-English sources^39,68^ through a combination of systematic searches on the Web of Science, Google Scholar and Google search engine, and opportunistic or targeted searches (e.g., contacting relevant experts). We increased the wealth of available data for invasive tree species through additional literature searches. We reviewed the literature published in English, Spanish and Portuguese until April 2022 using the words 'invas*' and 'econom*' as search terms, to which we added the scientific name of some known invasive tree species^31^ to obtain species-specific costs within their invaded ranges. This process resulted in 38 new cost entries. Each cost entry in InvaCost was standardized against a single currency (2017 United States dollars, hereafter $). Within the InvaCost database, a set of descriptor columns describes each cost in more detail to allow comparability across contexts and scales, including the taxonomy of invasive tree species, the temporal and geographic scales, the socioeconomic sectors affected, and the type of cost^68^.

### Data processing

We performed a series of filtering steps to obtain a subset of the InvaCost database containing only the costs of invasive trees. From the initial 13,553 cost entries available in the InvaCost database, we first selected only costs attributed to species or genera of plants that have a tree growth form (1044 cost entries). By adding the 38 new cost entries mentioned earlier, we obtained a set of 1082 records (Supplementary Table S1 online). Second, we removed costs which could not be converted to $ values from the original currency (n = 14), such as where official exchange rate information was unavailable from the World Bank Open Data^68^. Third, we removed cost entries without both starting and ending years simultaneously provided in the database, given they provide unreliable estimates of annual cost (n = 16). However, for those entries with one starting or ending year reported (i.e., with one or the other missing, not both), we conservatively assumed the cost spanned only one year (n = 4). This option, although potentially biasing the temporal distribution of the costs (note however that these costs were excluded for the temporal analysis; see section *Temporal trends*), allows the consideration of the entire reported cost of invasive alien trees meeting our selection criteria, which we describe below.

As costs in InvaCost are presented over different temporal scales (i.e., different lengths of time over which the cost occurred), we standardized our dataset so that each cost entry-realized over a single year, a period of less than a year, or a cost recurring over a series of years-corresponds to a single-year estimate, which is repeated over the number of years during which the cost occurred^68^. This means that the cost entries were ‘expanded’ without artificially inflating the aggregate cost. For example, an initial cost of $10 million over ten years would become, when expanded, ten entries of $1 million for each of the ten years. This process is crucial for examining the temporal trends in the development of costs, while minimizing temporal biases in cost reporting-which could arise from, for example, reporting a cost in a single year despite it spanning a longer period. This standardization on an annual basis was performed using the *expandYearlyCosts* function of the $$\texttt{invacost}$$ R package^69,70^. Further analyses were performed using this expanded version of the database.

### Cost descriptors

We used several key descriptors that are already present in InvaCost in the form of descriptive columns categorizing each reported cost (https://doi.org/10.6084/m9.figshare.12668570). We firstly distinguished costs based on their implementation, whereby ‘observed’ costs were actually incurred within the invaded habitat, whereas ‘potential’ costs were based on predictions over time or space within or beyond the species’ actual distribution area. Secondly, we distinguished costs based on the ‘reliability’ of the source material estimating/reporting them, whereby costs from officially pre-assessed material (e.g., peer-reviewed or official reports/documents), or material with documented, reproducible and traceable methods, were classified as of ‘high’ reliability, whereas all other costs were categorized as of ‘low’ reliability^69^. We used the ‘Method_reliability’ and the ‘Method_reliability_refined’ columns, with the latter, if provided, favored over the former in case of differing information (Supplementary Table S1 online). We first present full cost estimates, and then employ for further assessments a more focused and robust subset that includes only ‘observed’ and ‘highly reliable’ costs. Descriptors used to examine this subset of costs (called ‘robust subset’) included:

(i) taxonomy: the class, family, genus and species of invasive alien trees causing the cost;
(ii) geographic region: the continental region and country in which the cost was incurred. The map with spacial distribution of costs of invasive trees was generated using the rnaturaleath^71^ and the ggplot2 R packages^72^, where the International Standards Organization (ISO) 3-digit alphabetic country codes were assigned to InvaCost data using the countrycode R package^73^;
(iii) environment_IAS: the habitat where the invasive alien trees were identified (i.e., terrestrial, or semi-aquatic);
(iv) impacted sector: the activity, societal or market sector that was monetarily impacted by the invasive trees, which includes ‘Agriculture’, ‘Authorities-Stakeholders’ (governmental departments and/or official organizations such as conservation agencies, forest departments, associations; we note that this definition includes both authorities and stakeholders separately in a broad sense), ‘Public and social welfare’; ‘Environment’ and ‘Fishery’;
(v) type of cost: ‘Damage’ (i.e., costs of repairing damage, loss of resources), ‘Management’ (i.e., expenses on surveillance, prevention, control or eradication, etc.) and ‘Mixed’, (category used when reported costs were not easily distinguished between damage and management costs) and;
(vi) type of management cost: ‘pre-invasion’ (i.e., monetary investments for preventing successful invasions in an area-including quarantine or border inspection, risk analyses, biosecurity management, etc.), ‘post-invasion’ (i.e., money spent for managing invasions in invaded areas-including control, eradication, containment), ‘knowledge funding’ (i.e., money allocated to all actions and operations that could be of interest at all steps of management at pre- and post-invasion stages-including administration, communication, education, research, etc.) and ‘mixed’ (i.e., when costs include at least two of the previous categories and without possibility to disentangle the specific proportion of both). Detailed information on all descriptive variables can be found in the online repository of the InvaCost database (https://doi.org/10.6084/m9.figshare.12668570).

### Temporal trends

We examined the temporal development of invasion costs between 1960 and 2020, calculating annual costs^69^. We employed the $\texttt{invacost}$ R package through the *summarizeCosts* function. As explained above (*Data processing*), this temporal analysis removed entries for which the temporal duration was ambiguous, i.e., in the four entries where we had conservatively assigned a single year where the temporal scale was partly unspecified.

### Uses of alien trees

For those invasive alien trees with highly reliable costs we obtained their uses or reasons for the introduction from the Invasive Species Compendium of Centre for Agriculture and Biosciences International (CABI) database, Richardson & Rejmanek (2011) and the World Economic Plant database (WEP, National Plant Germplasm System GRIN-GLOBAL; https://npgsweb.ars-grin.gov/gringlobal/taxon/taxonomysearcheco, Accessed 13 Feb 2022). We followed the classification of uses of invasive alien trees determined by Richardson and Rejmanek (2011): commercial forestry; high quality furniture/wood; horticulture (ornamental, including coverage); agroforestry (including fodder), firewood and charcoal; food (including spices and medicines); stabilisation, erosion control and fertility improvement; and 'others' (including shade, biofuels and rubber). We evaluated the relationship between categories of uses of invasive alien trees and the costs of the invasive alien trees attributed to these uses. Thereby, we also examined whether species-specific costs were influenced by the numbers of distinct uses among individual invasive trees. For single species with multiple uses, we took the relative frequency of each use across all previously filtered InvaCost records for trees and used these relative frequencies to apportion each species' total costs into each use. For instance, if a species had 2 uses (use 1 and use 2) and the frequency of use 1 was 20%, and use 2 was 10%, the total cost X was considered as two thirds of use 1 and one third of use 2.

## Supplementary Information

Supplementary Information.

## Data Availability

All data generated and analyzed during this study are included in this published article (and its Supplementary Information files).
